# Differential Gene Expression and Infection Profiles of Cutaneous and Mucosal *Leishmania braziliensis* Isolates from the Same Patient

**DOI:** 10.1371/journal.pntd.0004018

**Published:** 2015-09-14

**Authors:** Eliza V. C. Alves-Ferreira, Juliano S. Toledo, Arthur H. C. De Oliveira, Tiago R. Ferreira, Patricia C. Ruy, Camila F. Pinzan, Ramon F. Santos, Viviane Boaventura, David Rojo, Ángelez López-Gonzálvez, Jose C. Rosa, Coral Barbas, Manoel Barral-Netto, Aldina Barral, Angela K. Cruz

**Affiliations:** 1 Departamento de Biologia Celular e Molecular e Bioagentes Patogênicos, Faculdade de Medicina de Ribeirão Preto, Universidade de São Paulo, Ribeirão Preto, São Paulo, Brasil; 2 Centro de Metabolómica y Bioanálisis (CEMBIO), Interacciones y Bioanálisis (UMIB), Universidad CEU San Pablo, Boadilla del Monte, Madrid, Spain; 3 Departamento de Química, Faculdade de Filosofia, Ciências e Letras de Ribeirão Preto, Universidade de São Paulo, Ribeirão Preto, São Paulo, Brasil; 4 Centro de Pesquisas Gonçalo Moniz (CPqGM)—Fundação Oswaldo Cruz (FIOCRUZ), Salvador, Bahia, Brasil; 5 Faculdade de Medicina da Universidade Federal da Bahia, Salvador, Bahia, Brasil; Liverpool School of Tropical Medicine, UNITED KINGDOM

## Abstract

**Background:**

Leishmaniasis is a complex disease in which clinical outcome depends on factors such as parasite species, host genetics and immunity and vector species. In Brazil, *Leishmania* (*Viannia) braziliensis* is a major etiological agent of cutaneous (CL) and mucosal leishmaniasis (MCL), a disfiguring form of the disease, which occurs in ~10% of *L*. *braziliensis*-infected patients. Thus, clinical isolates from patients with CL and MCL may be a relevant source of information to uncover parasite factors contributing to pathogenesis. In this study, we investigated two pairs of *L*. *(V*.*) braziliensis* isolates from mucosal (LbrM) and cutaneous (LbrC) sites of the same patient to identify factors distinguishing parasites that migrate from those that remain at the primary site of infection.

**Methodology/Principal Findings:**

We observed no major genomic divergences among the clinical isolates by molecular karyotype and genomic sequencing. RT-PCR revealed that the isolates lacked *Leishmania* RNA virus (LRV). However, the isolates exhibited distinct *in vivo* pathogenesis in BALB/c mice; the LbrC isolates were more virulent than the LbrM isolates. Metabolomic analysis revealed significantly increased levels of 14 metabolites in LbrC parasites and 31 metabolites in LbrM parasites that were mainly related to inflammation and chemotaxis. A proteome comparative analysis revealed the overexpression of *Lbr*PGF2S (prostaglandin f2-alpha synthase) and HSP70 in both LbrC isolates. Overexpression of *Lbr*PGF2S in LbrC and LbrM promastigotes led to an increase in infected macrophages and the number of amastigotes per cell at 24–48 h post-infection (p.i.).

**Conclusions/Significance:**

Despite sharing high similarity at the genome structure and ploidy levels, the parasites exhibited divergent expressed genomes. The proteome and metabolome results indicated differential profiles between the cutaneous and mucosal isolates, primarily related to inflammation and chemotaxis. BALB/c infection revealed that the cutaneous isolates were more virulent than the mucosal parasites. Furthermore, our data suggest that the *Lbr*PGF2S protein is a candidate to contribute to parasite virulence profiles in the mammalian host.

## Author Summary

Leishmaniasis is a critical public health problem worldwide. The clinical outcome of leishmaniasis depends on the infecting parasite species, host genetics and immune response and insect species. *Leishmania braziliensis* is a major etiological agent of cutaneous and mucosal leishmaniasis in Brazil. Fewer than 10% of *L*. *braziliensis*-infected patients with CL develop the mucosal form (a severe clinical manifestation). The small number of parasites in the mucosae increases the difficulty of obtaining clinical isolates, and parasite samples are frequently derived from individuals with different genetic backgrounds. Therefore, clinical isolates from cutaneous and mucosal sites from the same patient represent unique tools to understand parasite factors that contribute to disease outcome and pathogenesis. In this study, we investigated parasite factors involved in disease progression using two pairs of *L*. *(V*.*) braziliensis* isolates from mucosal (LbrM) and cutaneous (LbrC) sites of the same patient. In conclusion, the murine infection and proteome and metabolome data suggest that the differences between the cutaneous and mucosal isolates are mainly related to inflammation and chemotaxis. Our data also suggest that the *Lbr*PGF2S protein plays a role in parasite virulence in the mammalian host.

## Introduction

Leishmaniases, which are endemic in 98 countries (predominately in tropical and subtropical regions worldwide), represent a critical public health problem [1]. These diseases develop distinct clinical manifestations depending on the infecting *Leishmania* species, the composition of the sandfly vector saliva and the mammalian host’s genetic and immunological profile [2–4]. The output of infection varies widely; the symptomatic forms may be subdivided into tegumentary and visceral diseases. The tegumentary diseases may develop from mild (localized cutaneous leishmaniasis—LCL) to severe forms that include diffuse cutaneous leishmaniasis (DCL) and mucocutaneous disease (MCL) [5].

Approximately 20 species of *Leishmania* cause human infection, and tegumentary diseases may be caused by several species in different endemic countries. Each clinical form has been linked to one or a few species. For example, the *Leishmania* species from the *Viannia* subgenus (*Leishmania (V*.*) braziliensis*, *Leishmania (V*.*) guyanensis* and *Leishmania (V*.*) panamensis*), which are widely distributed in the Americas, are associated with not only CL but also MCL, which emerges in 5–10% of *L*. *braziliensis* infections [6–8]. In MCL, the oral and nasopharyngeal areas of the face are the most commonly affected and display tissue destruction characterized by intense inflammation and a low parasite load [7, 9]. The parasite factors determining the disease have been widely explored (but remain poorly understood) and it has been suggested that the *L*. *braziliensis* genotypes may be associated with specific disease manifestations [10].

The mechanisms that trigger the migration of *L*. *braziliensis* from the primary cutaneous site of infection to the facial mucosae are not understood. Various studies have suggested that migration of macrophages after infection may play a role in the dissemination of the parasite to other body regions, contributing to the development of MCL [11, 12]. However, the role of the parasite in the divergent behavior of host cells is unknown.

Although research using parasites collected from different patients are relevant to improving our understanding of mucosal disease, each patient has a distinct immunogenetic background and may respond to parasite infection differently. Therefore, clinical isolates from cutaneous and mucosal sites on the same patient represent unique tools that can be used to understand parasite factors that contribute to disease outcomes and pathogenesis.

Typically, mucosal lesions are diagnosed months or years after the primary cutaneous lesion. One study in an endemic area in Brazil evaluated 200 patients with CL by otorhinolaryngological examination and detected parasites in the nasal mucosae of six patients despite the absence of mucosal lesions [13]. There may be genetic differences between parasite populations isolated from cutaneous and mucosal sites. The genomes of the three *Leishmania* species *L*. *(L*.*) major*, *L*. *(L*.*) infantum* and *L*. *(V*.*) braziliensis* are highly conserved at the level of protein-coding gene content; however, the ample genetic plasticity of *Leishmania* is clear, suggesting that the key to understanding the diverse clinical manifestations, virulence and tropism of the parasite may be differential genome expression [10, 14, 15].

Here, we describe comparative expressed genome analysis and infectivity studies of two pairs of clinical isolates obtained from the primary site of infection and the mucosae of the same patient [13].

## Methods

### Parasites and culture conditions

*Leishmania (Viannia) braziliensis* were rescued from the biopsies of cutaneous lesions and mucosae of two patients from the endemic area of Jequié (Bahia/Brazil) with mucocutaneous leishmaniasis [13]. The isolates from cutaneous lesions were denoted LbrC^1^ (MHOM/BR/00/BA778) and LbrC^2^ (MHOM/BR/00/BA776), whereas parasites recovered from the mucosae were denoted LbrM^1^ (MHOM/BR/00/BA779) and LbrM^2^ (MHOM/BR/00/BA777); the numbers 1 and 2 refer to the respective patients. We have employed this simplified notation for the isolates throughout the text to facilitate comprehension. Wild type and transfectant promastigotes were maintained at 26°C in M199 medium supplemented as previously described [16]. Transfectants were maintained in liquid medium containing 6 x LD_50_ G418.

### PCR and RT-PCR

PCR was performed using GoTaq Flexi DNA Polymerase (Promega, Madison, USA) according to the manufacturer’s instructions. To confirm that the isolates belonged to the *Viannia* subgenus, genomic DNA (gDNA) extracted from the parasites was subjected to PCR using the primers 5’-CGGATCGCCCATGTACTC-3’ and 5’-GCATCGCAATAGTCCCACAT-3’ to amplify LbrM.23.0390 (RNAse III), which is specific to this subgenus [17].

All of the isolates were analyzed for the presence of the LRV virus (*Leishmania* RNA virus) by RT-PCR. cDNA was generated using reverse transcriptase (Invitrogen) and 1 μg of the extracted RNA according to the manufacturer’s protocol. PCR was performed using the primers LRV-for (5’-CGGTAGAGCATTAAGGGCTAGC-3’) and LRV-rev (5’-CGGCAGTAACCTGGATACAACC-3’), which amplify a genomic region conserved among all virus subtypes (kindly shared by MVG da Silva).

### Pulse Field Electrophoresis (PFGE)

Low-melting agarose plugs containing gDNA were prepared according to the protocol of Beverley *et al*. (1988) and Cruz *et al*. (1991) [18, 19]. The gDNA was fractionated in a 1% agarose gel by PFGE using two different programs. Program 1 was used to separate large chromosomes (4.5 V/cm, 144 h, 16°C, initial pulse: 360 s and final pulse: 800 s, 1x TBE buffer), whereas program 2 was used for smaller chromosomes (4.5 V/cm, 44 h, initial pulse: 50 s and final pulse: 120 s, 0.5x TBE buffer). The gels were stained with ethidium bromide for 90 min.

### Ethical statement

The use of mice and hamsters was approved by the Ethical Commission of Ethics in Animal Research (CETEA) at the Ribeirão Preto Medical School, University of São Paulo. They certified that Protocol n° 159/2011 (“Investigation of host-parasite interaction: exploring models of study of virulence and tropism”) is consistent with the ETHICAL PRINCIPLES IN ANIMAL RESEARCH adopted by Brazilian College of Animal Experimentation (COBEA) in 8/27/2012.

### Animal infection and parasite load

Hamsters were obtained from the facilities of the ANILAB Animais de Laboratório (Street Servidão Quatro, 292, Paulínia, SP, Brazil), and BALB/c mice were obtained from the institutional animal facility (Ribeirão Preto Medical School, USP, Ribeirão Preto, SP, Brazil). Mouse infection was conducted according to the guidelines of the Ethics Committee on Animal Experimentation from Ribeirão Preto Medical School, USP.

BALB/c mice and hamsters were infected *in vivo* with stationary-phase promastigotes (10^5^ viable parasites/10 μL PBS) by intradermal injection into the right ear (5 animals per experiment). Lesion progression was recorded once each week for six weeks by measuring ear swelling with a digital Vernier caliper using the non-infected contralateral ear as a control [20]. The parasite load was determined in the ear 4 weeks p.i. as described previously [21].

### *In vitro* infection

Peritoneal macrophages from BALB/c mice were maintained in RPMI medium supplemented with 10% (v/v) FBS. The cells (5x10^5^) were infected with late stationary promastigotes at a ratio of 10 parasites per macrophage for 4 h at 37°C. The infected cells were washed 3 times with incomplete RPMI 1640 (Life Technologies, Carlsbad, CA, USA) to remove non-internalized promastigotes and incubated at 5% CO_2_, 37°C for 0, 24 and 48 h. At the end of the assay, the infected macrophages were stained using the Diff Quick kit (LABORCLIN, Pinhais, Paraná, Brazil), and intracellular parasites were counted using a Leica DM500 microscope with a 100x objective. The parasite burden was verified by counting the number of infected macrophages in 300 cells (technical triplicates).

### *Ex vivo* analysis of IL-4 and IFN-γ

The production of IL-4 and IFN-γ cytokines was quantified using ELISA (Mouse IL-4 ELISA kit and Mouse IFN-γ ELISA kit- BD OptEIA) according to the manufacturer’s protocol (BD Biosciences, San Diego, CA, USA). Cell suspensions were prepared from the lymph nodes and spleens of BALB/c mice infected for 4 weeks with LbrC^1^, LbrM^1^, LbrC^2^ and LbrM^2^ promastigotes. Overall, 5x10^6^ cells/mL were plated per well in 24-well tissue culture plates and stimulated with 40 μg/mL *L*. *braziliensis* particulate antigens (SLA) as previously described [22].

### Two-dimensional gel electrophoresis (2DE) and protein identification

The protein extracts of logarithmic phase promastigotes (5x10^8^ parasites) were obtained by precipitation with trichloroacetic acid, and the samples were subjected to 2D gel electrophoresis using Immobiline DryStrip gels (13 cm/4-7 pI) (GE HealthCare, Piscataway, New Jersey, USA). Protein extraction and 2D electrophoresis were performed as described previously [23, 24]. A comparative analysis of the digitized proteome maps of the LbrC and LbrM isolates was performed using ImageMaster platinum v6.0 software (GE Healthcare). Genes were considered differentially expressed when the spot intensity was increased 1.5-fold between isolate pairs. All of the analyses were performed in biological triplicate, and we used a two-sample t-test to compare the differentially expressed spots. The peptides were identified by MALDI-TOF/TOF mass spectrometry at the Center for Protein Chemistry (University of São Paulo, Ribeirão Preto) and analyzed using the MASCOT program (Version 2.2.04) and the GeneDB genome of *L*. *braziliensis*.

### Construction of overexpression plasmid and transfection

The LbrPGF2S CDS flanked by 800 bp was amplified from the gDNA of *L*. *(V*.*) braziliensis* strain MHOM/BR/75/M2904 using the primers 5’FLR-LbrPGF2S_NheI-for (5’-GCTAGCAGGTGTGCTACAGGTAAGGAAGC-3’) and 3’FLR-LbrPGF2S_HindIII_XbaI-rev (5’-TCTAGAAAGCTTGCATGAAGAAGAGGGTCCAG-3’) and cloned into the pGEM-T easy vector (Promega) according to the manufacturer's instructions. The 2.3-kb fragment resulting from digestion with the *Nhe*I and *Hin*dIII enzymes containing LbrPGF2S CDS and its 3’ and 5’ UTRs [25] was subcloned into the *pXNEO* vector [26] for overexpression in *Leishmania*.

Promastigotes were transfected with the *pX63NEO* and *pX63NEO-LbrPGF2S* plasmids by electroporation. Transfectants were selected in semi-solid M199-agar medium in the presence of the G418 antibiotic (Sigma, St. Louis, MO). The G418 LD_50_ was determined for each isolate, and four- or six-fold LD_50_ was used. Promastigotes were maintained at 26°C in M199 medium supplemented as previously described [16]. For *in vitro* infection, the mutant promastigotes were cultivated in a liquid medium containing six-fold G418 LD_50_.

### Western blotting

Total protein extracts were obtained from 1x10^7^ promastigotes by precipitation with trichloroacetic acid (a similar extraction was used for the 2D analysis). The proteins were fractionated by 12% SDS-PAGE and blotted onto Hybond ECL membranes in a TE22 mini transfer unit (both from GE Healthcare) [23]. The membranes were blocked in 3% BSA blocking buffer for 1 h, incubated with chicken anti-*Lbr*PGF2S (1:10000) (produced by our group) for 1 h, washed, and incubated with peroxidase-conjugated anti-chicken IgY (1:800,000) (Sigma) for 1 h at room temperature. Antigen-antibody interactions were detected using an ECL kit (GE Healthcare); chemiluminescence was visualized using an ImageQuant LAS 4000 (GE Healthcare).

### Genomic DNA library sequencing and analysis

Genomic DNA from each isolate (LbrC^1^, LbrC^2^, LbrM^1^ and LbrM^2^) was sequenced from paired-end DNA libraries constructed with the Nextera XT DNA Sample Preparation kit. The Illumina MiSeq platform (FMRP-USP) was used for library sequencing (150-nt long reads). Read quality, length and number were verified using the FastQC tool (http://www.bioinformatics.babraham.ac.uk/projects/fastqc/); reads with an average quality value of less than 30 were removed. The Illumina adapter was removed using cutadapt software (version 1.4.1) (http://journal.embnet.org/index.php/embnetjournal/article/view/200).

The reads were aligned against the LbrM2903 version 8.0 reference genome (S1 Table) available at TriTrypDB [27] using BWA [28] (Version 0.7.10-r789) to generate alignments in the *sam* format. The BWA-MEM algorithm with default values was applied for 150-bp Illumina reads. SAMtools [29] was used to convert the sam files into binary format and sort, index and count the reads from each chromosome in bam files. These bam files were visualized with Artemis [30]. The chromosome somy in each library was calculated independently according to the method of Zhang *et al*. [31].

### Metabolite extraction of *Leishmania* for metabolomic fingerprinting

For each compared group, LbrC and LbrM, seven biological replicates were collected in the late-log phase of promastigote culture. Before metabolite extraction, metabolism was quenched by shaking the 10-mL culture bottle in an ethanol/dry-ice bath for 40 sec. For HPLC-MS and CE-MS, 4x10^7^ promastigotes were centrifuged at 2,000 x *g* for 10 min at 4°C, washed 3 times in cold (4°C) PBS (137 mM NaCl, 8 mM Na_2_HPO_4_, 2.7 mM KCl, and 1.5 mM KH_2_PO_4_; pH 7.0) and lysed in 450 μL of cold (4°C) CH_3_OH/H_2_O (4:1, v/v). The cells were mechanically disrupted by 3 freeze/thaw cycles in liquid N_2_, followed by lysis for 10 min at 50 Hz in a TissueLyser LT (Qiagen) with glass beads (50 mg, 425–600 μm, Sigma). The cellular debris was removed by centrifugation at 15,700 x *g* at 4°C for 10 min. For HPLC-MS, 200 μL of clarified supernatant was transferred into a glass vial and submitted to HPLC-MS analysis. For CE-MS, 200 μL of clarified supernatant was transferred into a new tube, dried and re-suspended in 200 μL of milli-Q water.

For GC-MS, metabolites were extracted by the same process in 350 μL of CH_3_OH/CHCl_3_/H_2_O (3:1:1, v/v/v) at 4°C. The supernatant (200 μL) was clarified by centrifugation and evaporated to dryness in a SpeedVac at 30°C. Next, 10 μL of *O*-methoxyamine hydrochloride (15 mg/mL in pyridine) was added to each GC vial, mixed vigorously for 5 min using a vortex FB 15024 (Fisher Scientific, Madrid, Spain), and incubated in darkness at room temperature for 16 h for methoximation. Then, 10 μL of BSTFA (N,O-bis(trimethylsilyl)trifluoroacetamide) with 1% TMCS (v/v) (trimethylchlorosilane) was added, and the vials were vortexed for 5 min and incubated for 1 h at 70°C for the silylation reaction. Finally, 100 μL of heptane containing 10 mg/mL C18:0 methyl ester (internal standard) was added, and the samples were vortexed. Two blank samples were prepared following the same extraction and derivatization procedures.

Quality controls (QCs) were independently prepared for each technique by pooling equal volumes of each sample. The controls were analyzed at the start of each analysis to reach system equilibration and throughout the run to provide a measurement of the system’s stability and the reproducibility of the sample treatment procedure.

### Metabolomic fingerprinting by HPLC-MS, CE-MS and GC-MS

Considering the large chemical diversity of metabolites, the samples were analyzed by HPLC-MS, CE-MS and GC-MS to ensure wide coverage encompassing hydrophobic, hydrophilic, acidic, basic and neutral molecules. The HPLC-MS, CE-MS and GC-MS instrumentation and settings for metabolomic analysis were as previously described by Canuto *et al*. [32].

### Metabolomic data treatment and statistical analysis

Background noise and unrelated ions were removed from the resulting data files (HPLC-MS and CE-MS) using the Molecular Feature Extraction (MFE) tool in Mass Hunter Qualitative Analysis software (B.05.00, Agilent). Primary data treatment (filtering and alignment) was performed using Mass Profiler Professional software (B.02.01, Agilent). Data treatment for GC-MS analysis was conducted through compound identification using the Fiehn retention time locked (RTL) library and the National Institute of Standards and Technology mass spectra library with MSD ChemStation software (G1701EA E.02.00.493, Agilent) and a correct assignment based on the coincidence of the retention time and the spectrum profile [33]. For all of the analytical platforms, features that did not appear in at least 50% of the QCs with a coefficient of variation less than 30% were excluded from the analysis. The metabolic profiles were analyzed by principal component analysis (PCA) (S1 Fig). We considered a metabolite to have a differential profile between LbrC and LbrM only in the following situations: (*i*) when there was a statistically significant differential abundance in the samples from the two phenotypes (Student’s *t*-test, *p* value < 0.05); and (*ii*) when the metabolite was consistently detected in 100% of the biological replicates per group. The accurate masses representing statistically significant differences were searched in MassTrix [34] and CEU Mass Mediator (http://ceumass.eps.uspceu.es/mediator/). The heatmap was designed using MetaboAnalyst (v. 2.0) [35].

### Accession numbers

All sequence information was deposited in GenBank under bioproject ID PRJNA292004. DNA sequencing data can be accessed from the SRA database using accession no. SRP062173.

## Results

### *L*. *braziliensis* cutaneous and mucosal isolates do not exhibit major genomic differences

**Fig 1:**
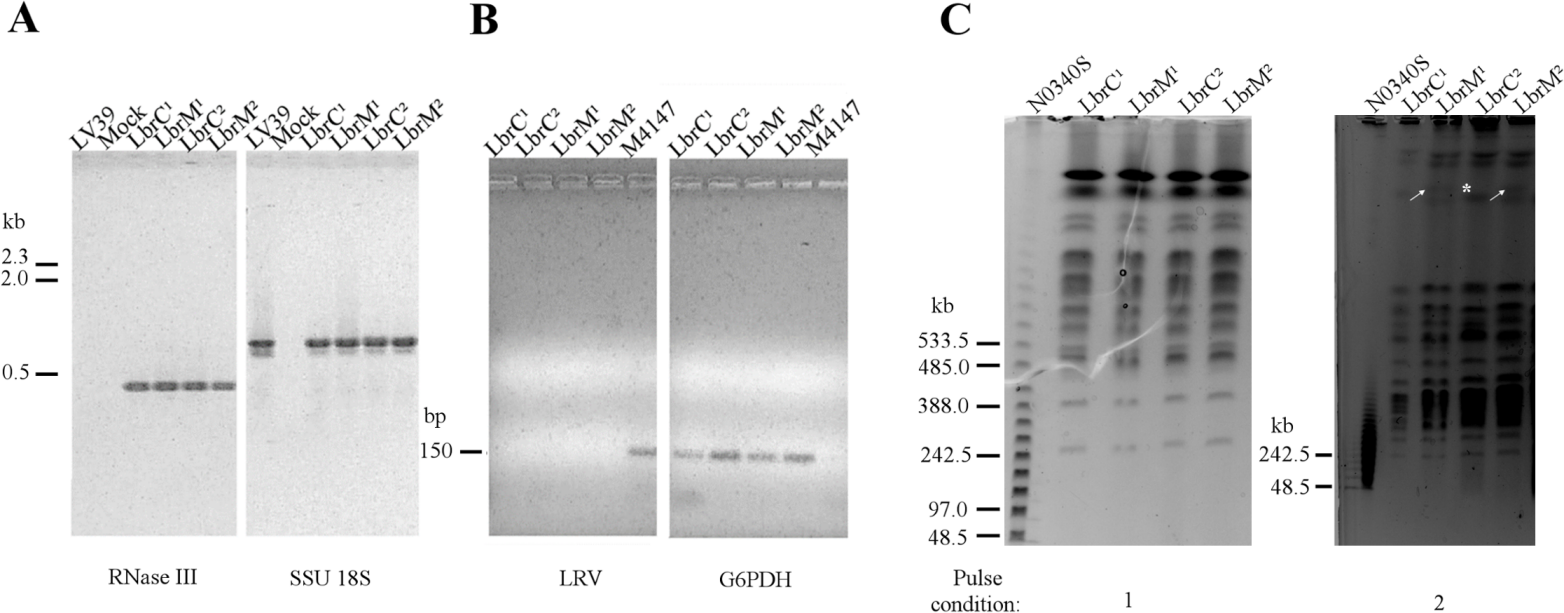
Molecular characterization of the LbrC and LbrM clinical isolates. **(A)** Agarose gel-fractionation of the PCR products from DNA extracted from LbrC^1^, LbrM^1^, LbrC^2^ and LbrM^2^ parasite cultures verified that the isolates belong to the *Viannia* subgenus. The *Viannia* subgenus was identified by PCR using primers for the RNase III domain gene (LbrM.23.0390) and 18S rDNA (SSU) gene as a DNA quality control. The LV39 strain [*Leishmania (Leishmania) major*] and a mock reaction (PCR reaction without genomic DNA) were used as negative controls. **(B)** PCR analysis of DNA extracted from LbrC^1^, LbrM^1^, LbrC^2^, LbrM^2^ and M4147 parasite cultures fractionated in agarose gels demonstrated that the *Leishmania* virus (LRV) was not amplified by RT-PCR from cutaneous and mucosal isolates. cDNA from *Leishmania Viannia guyanensis* (M4147 strain), which harbors the virus, was used as a positive control, and G6PDH primers were used as a positive control for *Leishmania* DNA. **(C)** Molecular karyotypes of parasites rescued from cutaneous and mucosal sites (LbrC^1^, LbrM^1^, LbrC^2^ and LbrM^2^), as determined by pulsed-field gel electrophoresis (PFGE). The electrophoresis conditions are described in the Methods section. Numbers 1 and 2 refer to electrophoresis programs. Marker: N0340S (NE BioLabs). The gels were stained with ethidium bromide. White arrows indicate bands observed only in the LbrM isolates. * highlights the LbrC^2^ band with a doubled signal intensity compared to LbrM^2^.

Paired clinical isolates were recovered from two patients in a leishmaniasis endemic area in Brazil where *Leishmania (Leishmania) amazonensis* and *Leishmania (Viannia) braziliensis* species are responsible for CL, according to the *Manual for Surveillance of American Integumentary Leishmaniasis* [36]. All of the isolates were subjected to PCR using primers to amplify a fragment of a *Viannia*-specific gene (LbrM.23.0390, RNase III domain gene). Reactions using parasite genomic DNA from all four isolates amplified the control DNA (SSU 18S) and the RNase III domain gene, confirming the isolation of the *Leishmania (V*.*) braziliensis* species (Fig 1A). We used a *Leishmania* (*Leishmania) major* strain as a negative control for the *Viannia*-specific fragment, from which no amplification was obtained (LV39 lane).

Based on a recent study suggesting a positive correlation between the presence of LRV virus in *Leishmania (Viannia)* spp. and metastatic behavior [37], we searched for the presence of LRV RNA in all four isolates. We could not detect the presence of the virus by RT-PCR in the LbrC and LbrM isolates; *L*. *(V*.*) guyanensis* (M4147 strain) was used as a positive control (Fig 1B). This result suggests that the metastatic behavior of the LbrM isolates is not associated with LRV.

To investigate possible genomic differences between the LbrC and LbrM isolates recovered from the same patient, agarose-embedded genomic DNA was fractionated by pulsed field gel electrophoresis (PFGE). Using two different pulse conditions to fractionate small/medium or large chromosomes, we observed a similar karyotype, with the exception of one extra band representing a large chromosome that was exclusively present in both LbrM isolates (Fig 1C). Comparative analysis of the signal intensity of this ‘novel’ band in the LbrM^2^ karyotype with the corresponding region of the LbrC^2^ (marked with *) is suggestive of one allele size increment. Overall, the karyotype analysis revealed high similarity among the four isolates, suggesting that all belong to the same strain.

**Fig 2:**
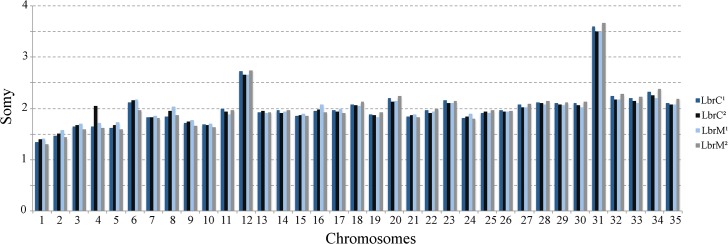
Chromosome somy of the LbrC and LbrM isolates. The median coverage for a haploid allele of a chromosome was calculated. The median coverage of each chromosome was divided by the haploid chromosome coverage to obtain the somy of the individual chromosomes [31].

To confirm the similarity of genomic content and investigate possible chromosome somy changes, we subjected both of the LbrC/LbrM pairs to NGS genomic sequencing. As shown in Fig 2 and S2 Table, no consistent differences between paired isolates were detected, confirming the lack of major genomic changes.

### Cutaneous isolates are more virulent during *in vivo* infection

**Fig 3:**
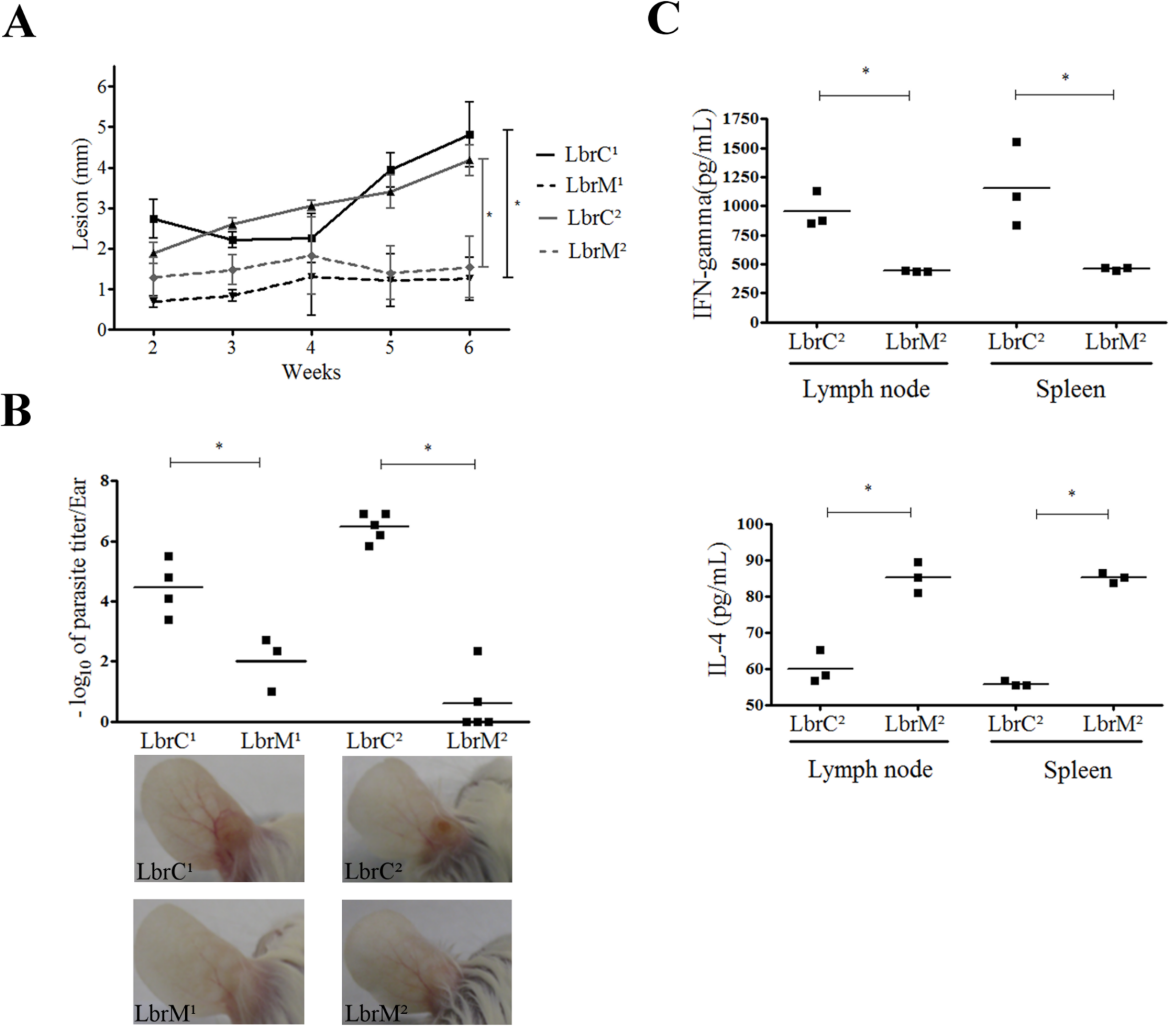
LbrC and LbrM isolates exhibit distinct virulence in BALB/c mice. **(A)** The panel represents the lesion development in hamsters (*Mesocricetus auratus*) after infection with the isolates. Ear thickness was measured with a Mitutuyo digital caliper for six weeks. Each point represents the mean lesion size (±SEM) of five animals per group. **(B)** The graph represents the parasite load in the ear of BALB/c mice after a four-week infection with the isolates by limiting dilution assay. Three or five mice were used per group. The *lower panel* shows representative images of ear lesions after 4 weeks of infection. **(C)** Quantification of IFN-γ and IL-4 cytokines released by lymph node and spleen cells after one month of infection with the LbrC^2^ and LbrM^2^ isolates following stimulation with SLA for 72 h. Three mice were included in each group. *p<0.05 (two-tailed t-test).

We investigated whether the parasites rescued from the primary site of infection versus those rescued from the mucosae of the same patient had a different infection profile. *In vivo* infection in hamsters and BALB/c mice revealed that both LbrC isolates displayed a more severe clinical manifestation. Hamsters infected with the LbrC isolates produced larger lesions than hamsters infected with the LbrM isolates (Fig 3A). BALB/c mice were used to evaluate the virulence of the isolates. The parasite burden was increased at the inoculation site (ear) of LbrC-infected animals compared to LbrM-infected mice at 4 weeks p.i. (Fig 3B). Therefore, both parasite load and lesion size measurement revealed a different murine infection profile between the LbrC and LbrM isolates. Also, significantly increased IL-4 levels were detected in cell cultures derived from the lymph nodes and spleens of LbrM^2^-infected mice compared with cultures from mice infected with LbrC^2^ (Fig 3C). In contrast, LbrC^2^-infected cells released more IFN-γ than cells infected with LbrM^2^. In both cases, the cells were stimulated with the same antigens (SLA).

### Cutaneous and mucosal isolates present clear-cut differences in their metabolomes

To obtain an overview of the global physiological differences between LbrC and LbrM, we subjected one of the pairs (LbrC^1^ and LbrM^1^) to a comparative metabolomic analysis. The tight cluster of QCs (quality controls) in the unsupervised PCA model scatter plots for HPLC-MS, CE-MS and GC-MS confirmed technical reproducibility (S1 Fig).

S3 Table summarizes the metabolomic data obtained from the different techniques, including the monoisotopic mass, retention time, percentage change, *p* value and biological role.

**Fig 4:**
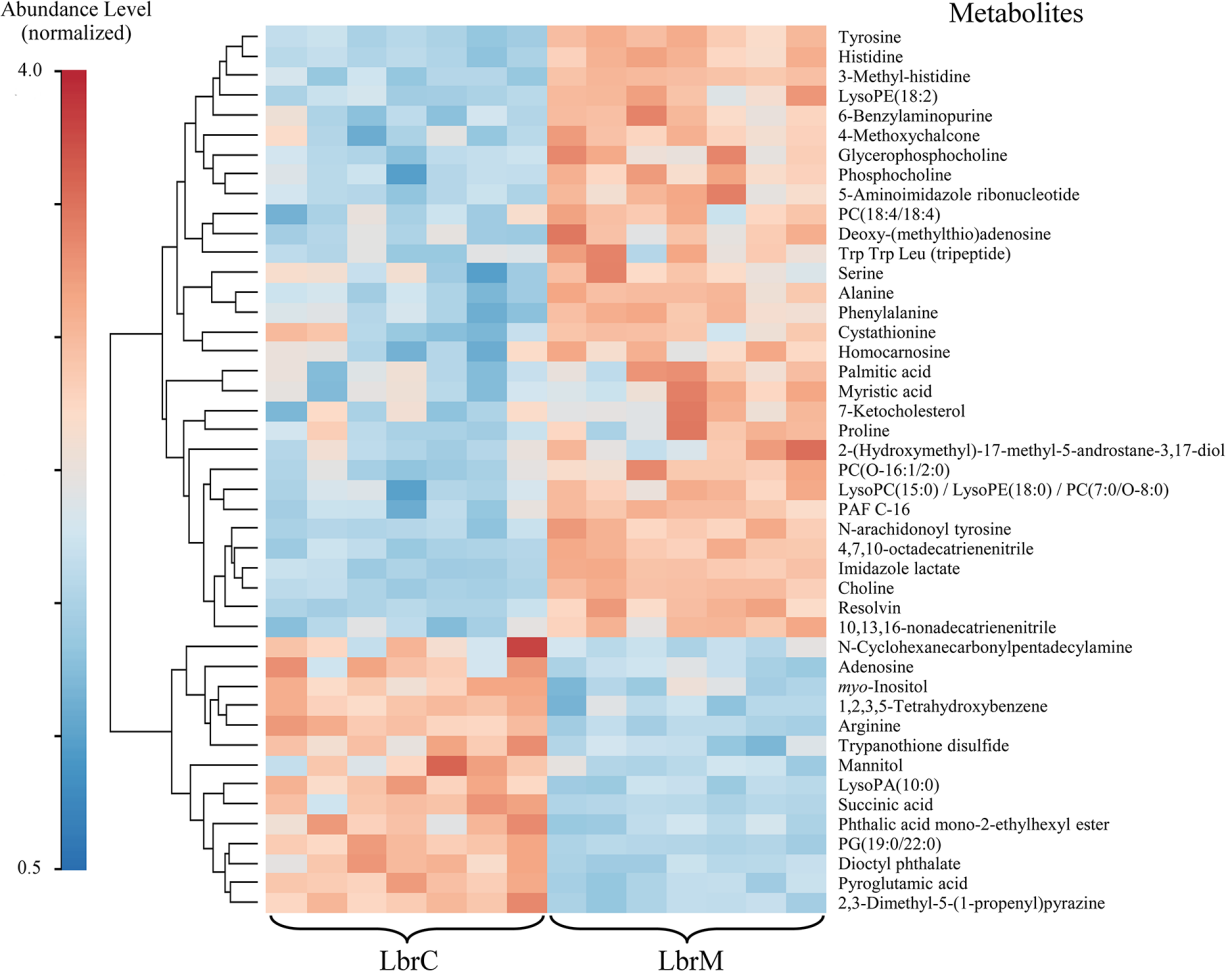
Hierarchical Clustering Heatmap of biologically relevant metabolites in LbrM and LbrC. The color code indicates the metabolites abundance. To enable the comparison of data obtained from HPLC-MS, CE-MS and GC-MS the metabolite abundance was normalized. The MetaboAnalyst (v. 2.0) website was used to normalize the data and to generate the figure. The normalization procedure consisted of mean-centering and division by the standard deviation of each variable. The lines in the heatmap represent the relative abundance of metabolites across the samples of the two compared groups, LbrC and LbrM; each metabolite is indicated on the right side of the figure. The columns corresponding to the LbrM and LbrC groups are indicated at the bottom. Each of the seven columns corresponds to one biological replicate (seven per group). To the left-hand side of the figure, a scale indicates the color code relative to the normalized metabolite abundance (ranging from 0.5 up to 4.0).

A heat map was constructed to visualize differences in the LbrC and LbrM metabolomes (Fig 4). The heatmap revealed important differences in the intracellular concentration of metabolites. The hierarchically clustered heat map revealed 31 metabolites whose levels were significantly decreased in the LbrC parasites and 14 metabolites that were decreased in the LbrM parasites. The metabolome differences indicated a clear metabolomic dichotomy in the parasite population from the cutaneous site versus the mucosae of the same individual. Most of the metabolites that were present at different levels in the compared samples were related to inflammatory processes.

Several phospholipids and related metabolites, such as choline, saturated fatty acids (myristic and palmitic acids) and ketocholesterol, were identified among the up-regulated metabolites in the LbrM samples. Others metabolites, such as phosphatidic acid (PA) and phosphatidylglycerol (PG), were detected at lower levels in LbrM (Fig 4).

The analysis also revealed differences in “amino acids and derivatives” between the compared lines. Purine metabolism differed between the two lines, with some metabolites up-regulated or down-regulated in the metastatic line (Fig 4). Chalcone levels were higher in LbrM, whereas trypanothione disulfide (a reduced form of trypanothione) levels were lower (Fig 4). Other metabolites from different chemical classes that participate in pathways related to purine and polyamine metabolism and/or redox routes were expressed at different levels in the LbrC and LbrM samples.

### *Lbr*PGF2S and HSP70 are up-regulated in cutaneous isolates

As part of the global comparative analysis of the LbrC and LbrM isolates, we investigated possible modifications of gene expression between parasites from mucosal and cutaneous sites through a comparative analysis of proteome profiles. The proteomes were evaluated by protein fractionation through two-dimensional gel electrophoresis, followed by identification of differentially expressed spots by mass spectrometry.

We considered a differential expression positive if the differences in spot signal intensities were greater than 1.5-fold between the compared proteomes. In each replica of the fractionated proteome, we detected 477, 488 and 525 spots for LbrC^1^, 358, 351 and 434 spots for LbrM^1^, 448, 440 and 345 spots for LbrC^2^ and 406, 346 and 387 spots for the LbrM^2^ samples. The percentage of corresponding (matched) spots among the LbrC^1^/LbrM^1^ and LbrC^2^/LbrM^2^ pairs was satisfactory (61.10% and 70.49%, respectively).

Differentially expressed spots were excised from the gels and subsequently analyzed by mass spectrometry. Twenty-four polypeptides were identified under these conditions in the comparative analysis of the LbrC^1^ and LbrM^1^ protein extracts, and 23 polypeptides were identified in the analysis of the protein extracts of LbrC^2^ and LbrM^2^ (S4 Table). Among these, only LbrM.31.2410 (prostaglandin f2-alpha synthase- *Lbr*PGF2S) and LbrM.28.2990 (HSP70, putative) were consistently over-represented in both cutaneous isolates (LbrC^1^ and LbrC^2^) and less abundant (or undetectable) in both mucosal isolates (LbrM^1^ and LbrM^2^).

### Ectopic expression of *Lbr*PGF2S increases parasite virulence during infection *in vitro*

Both the proteome and metabolome data suggested that PGF2S was an important target warranting further investigation. Thus, we generated LbrC^1^ and LbrM^1^ parasites overexpressing *Lbr*PGF2S to confirm the correlation between *Lbr*PGF2S levels and virulence. The gene was inserted into *pX63NEO* and expressed ectopically. *Lbr*PGF2S overexpression was confirmed by Western blotting using a polyclonal anti-*Lbr*PGF2S antibody (S2 Fig).

**Fig 5:**
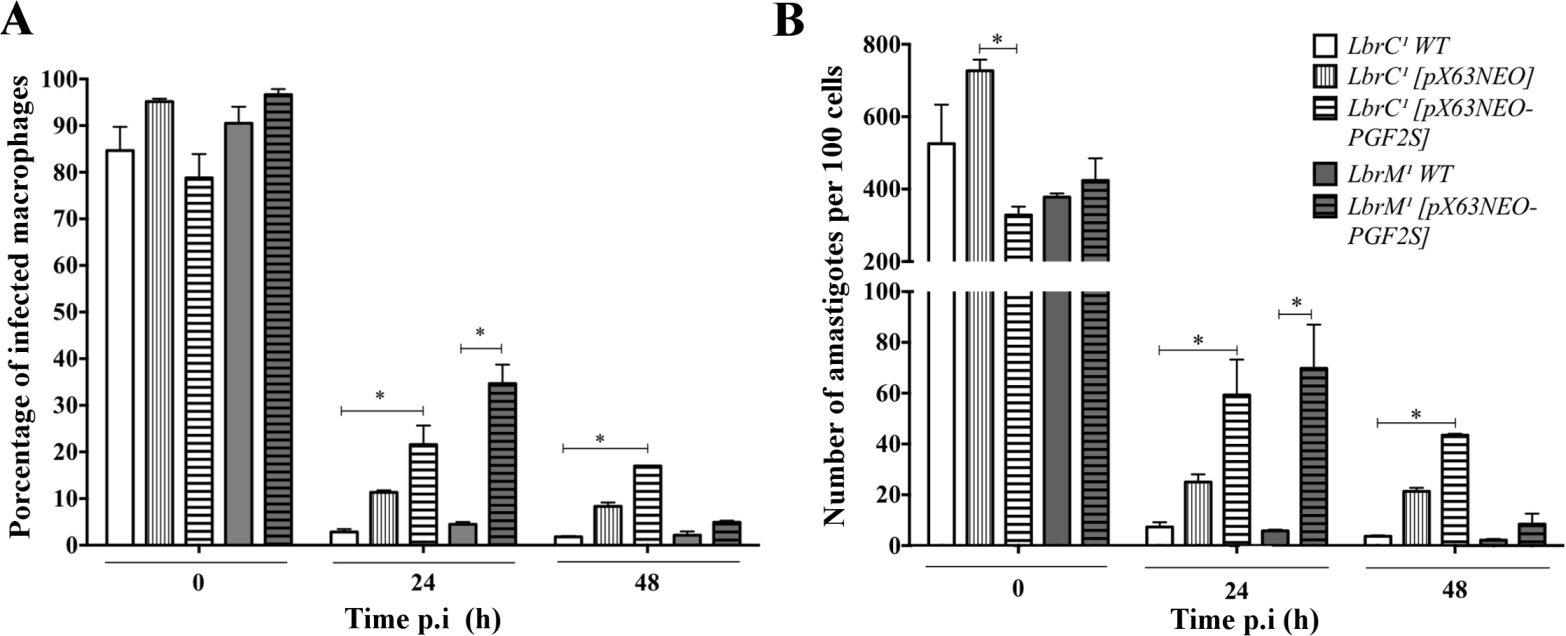
Ectopic overexpression of *Lbr*PGF2S increases the infection index *in vitro*. **(A)** Peritoneal macrophages from BALB/c mice were infected with Lbrc and LbrM transfectants and the wild type strain. At 0 h, 24 h and 48 h post-infection, the cells were stained, and 600 cells were counted. Each bar represents the average and SD of three replicates. *p<0.05 (Student's t test). **(B)** Number of amastigotes in the macrophages.

Infection of peritoneal macrophages from BALB/c mice with the *wild type* gene and transfectants resulted in average macrophage infection rates of 84%, 95%, 78%, 90% and 96% (t0 post-infection) for LbrC^1^ *wild type*, LbrC^1^ *[pX63NEO]*, LbrC^1^ *[pX63NEO-PGF2S]*, LbrM^1^ *wild type* and LbrM^1^ *[pX63NEO-PGF2S]*, respectively, indicating that the percentage of internalization during early infection did not vary significantly. Nevertheless, after 24 h, the infection index decreased to 2.8% and 4.5% for LbrC^1^ *wild type* and LbrM^1^ *wild type*, respectively, and to 21.6% and 34.6% for the *Lbr*PGF2S-overexpressing transfectants. Thus, the *Lbr*PGF2S-overexpressing parasites exhibited a significantly increased infection percentage 24 h p.i. This difference persisted until 48 hours p.i. for LbrC^1^ *[pX63NEO-PGF2S]* (Fig 5). Immediately before the *in vitro* infection experiments, the overexpression of *Lbr*PGF2S was confirmed by Western blotting (S2 Fig).

The number of amastigotes within macrophages decreased at 24 h and 48 h p.i. for all groups. Nevertheless, the differences between the wild type and *LbrPGF2S*-overexpressing infections persisted at these time points. The average numbers of intracellular amastigotes were 7.3, 5.8, 59.3 and 69.8 per macrophage (300 cells counted) for infections with LbrC^1^ *wild type*, LbrM^1^ *wild type*, LbrC^1^ *[pX63NEO-PGF2S]* and LbrM^1^ *[pX63NEO-PGF2S]*, respectively. Both of the overexpressor transfectants produced a significant increase in amastigotes inside macrophages compared with the wild type; this increase was most apparent at 24 h p.i.

## Discussion

Here, we used a powerful tool to explore parasite factors involved in the pathogenesis of leishmaniasis. We demonstrated that significant differences in the proteome, metabolome and parasite virulence may emerge from two subpopulations of the same *L*. *braziliensis* strain collected from different tissues in the same human host. We propose that parasite factors other than the presence of the *Leishmania* RNA virus (LRV) are involved in specific manifestations in tegumentary leishmaniasis when the immunogenetic background is the same. The data presented here are the first to suggest that the enzyme *Lbr*PGF2S may participate in the virulence profile in the host. Our findings were reproducible for two “same-host” pairs isolated from two different patients.

We demonstrated that these *L*. *(V*.*) braziliensis* isolates consistently exhibited differences in the expressed genome, metabolome and pathogenesis *in vivo*. We determined that the four isolates possessed similar molecular karyotypes and therefore likely originated from a single circulating strain. In addition, complete genome sequencing did not reveal significant somy differences between the LbrC and LbrM isolates. The inclusion of these samples in the *Viannia* subgenus was confirmed by PCR based on the presence of a domain from the Dicer-like gene that is an integrant of the RNA interference pathway exclusive to the *Viannia* subgenus [17].

The lack of LRV in the LbrC and LbrM isolates is relevant in light of the reported correlation between the presence of LRV and the severity of clinical manifestations of tegumentary leishmaniasis. The parasite virus triggers host macrophage recognition, promoting inflammation and modifying the immune response during infection, thereby conferring parasite fitness advantages within the host cell [37]. Therefore, it is possible to speculate that differences in the expressed genomes of the parasites found at the infection site and those isolated from the nasal mucosae are partially responsible for the diverse pathogenesis of LbrC and LbrM isolates.

*In vivo* infection experiments revealed that the analyzed parameters—i.e., parasite load and lesion progression—differed between the LbrC and LbrM isolates. Hamsters and BALB/c mice infected with the LbrM isolates exhibited smaller lesions and lower parasite burdens at the site of primary infection and the draining lymph nodes compared to animals infected with LbrC. These findings are in accordance with those of Jara *et al*. [9], who previously have demonstrated a lower parasite burden in the mucosae compared to that at cutaneous sites. However, we must emphasize that the parasite populations analyzed in this study were recovered from patients during the acute phase of infection, whereas the mucosal isolates from the study performed by Jara *et al*. were recovered from a chronic infection.

The differences in IL-4 and IFN-γ levels suggest that the LbrC and LbrM isolates stimulated different host cell responses in BALB/c mice. Also, higher levels of IFN-γ in the supernatants of cells infected with LbrC^2^ are consistent with the increased lesions observed in hamsters infected with LbrC^2^. Nevertheless, to pursue a robust characterization of the immune response associated to mucosal or cutaneous isolates, it will be necessary to quantify other Th1 or Th2 pattern-specific cytokines.

We investigated the features of the phenotypic expression that could be associated with the pathogenesis of the parasite at the mucosae. The comparison of the proteomes and metabolomes of the paired strains revealed interesting features.

The observed metabolome differences indicate a clear dichotomy between the parasite populations resting at the cutaneous site versus the population localized to the mucosae of the same individual. Many of the metabolites that significantly differed between isolates may affect several cellular processes, and an investigation of the relevant metabolic pathways is needed to understand the role of these metabolites in pathogenesis. However, the metabolome profiles of LbrC and LbrM indicate that the differences in pathogenesis involve the differential production of metabolites related to inflammation and chemotaxis.

The complex interaction of molecules that determine the migration of *L*. *braziliensis*-infected host cells from the primary lesion site to the mucosal regions remains undetermined. However, previous studies have demonstrated that *Leishmania* promastigotes release chemotactic factors that regulate cell migration and the activation of the innate immune system at the primary site [38, 39]. In this study, we used proteomic and metabolomic analyses to obtain a more global understanding of the physiological differences between the LbrC and LbrM isolates and to identify chemotactic networks that guide or contribute to the differential pathogenesis of *L*. *braziliensis*.

Some of the fatty acids that were elevated in LbrM (i.e., myristic and palmitic acids) may affect the inflammatory reaction, playing a key role in parasite tropism or exert pro-inflammatory activities. Others may affect protein anchoring to membranes (which is critical for the recognition and attachment of parasites to host cells) or signal transduction pathways [40–43].

Resolvins were markedly elevated in LbrM. Resolvins are lipid mediators that stimulate pro-resolving mechanisms during sepsis [44]. Resolvins may decrease the migration of inflammatory and dendritic cells [44, 45], suppress NF-κB activation [46], enhance phagocytosis and anti-inflammatory cytokine production and stimulate host-protective actions in inflammatory responses [47].

Several metabolites (i.e., phosphatidylcholines, phosphatidylethanolamines and their derivatives) involved in phospholipid synthesis were up-regulated in the LbrM samples. Parasites rely on a complex system of uptake and synthesis mechanisms to obtain lipids at different life stages [48], and lipid metabolism is crucial for the production of factors related to pathogenesis. The increased levels of phospholipids suggest that the Kennedy pathway [49] could be more active in the LbrM strain. However, a targeted metabolomics approach is needed to more closely evaluate the levels of Kennedy metabolites. The platelet-activating factor (PAF) was also increased in LbrM; PAF is involved in a variety of inflammatory processes such as vascular permeability, oxidative burst, chemotaxis and activation of leukocytes, stimulation of arachidonic acid metabolism [50] and cellular differentiation and infectivity in *Trypanosoma cruzi* [51].

Cyclohexanecarbonylpentadecylamine and phthalic acid mono-2-exthylhexyl ester, metabolites related to arachidonic acid metabolism, were decreased in LbrM [52]. The cellular starting material for prostaglandin biosynthesis is usually membrane phosphatidylinositol, whereas prostaglandins are predominantly synthesized from arachidonic acid, which is also a prostaglandin synthase substrate in protozoan parasites that release prostaglandins [53].

Chalcone, a metabolite that can affect local inflammatory responses and the prostaglandin synthesis pathway, was up-regulated in LbrM. Chalcones may suppress the mitogen-activated protein kinase (MAPK) pathway, thereby inhibiting pro-inflammatory mediators such as nitric oxide (NO), prostaglandin E(2), tumor necrosis factor-alpha (TNF-alpha) and the production of reactive oxygen species [54, 55]. The combination of the up-modulation of prostaglandin biosynthesis metabolites in LbrC and the up-modulation of inhibitory metabolites in the prostaglandin pathway (4-metoxychalcone) in LbrM suggests that prostaglandin metabolism could play a role in *L*. *braziliensis* pathogenesis.

Interestingly, the proteome comparative analysis reinforced the potential relevance of the prostaglandin pathway to the pathogenesis of *L*. *braziliensis* infection. The proteome analysis enabled the detection of two proteins with matching differential expression patterns between different host isolates from cutaneous site in the two patients: prostaglandin F2 alpha synthase (LbrM.31.2410) and putative HSP70 (LbrM.28.2990). These proteins were uniquely expressed or over-expressed in the LbrC^1^ and LbrC^2^ samples, respectively. Remarkably, survival of *L*. *infantum* promastigotes of nitric oxide donor exposure [56] has been associated with HSP70 and PGF2S overexpression and increased *in vitro* infectivity. These results support the hypothesis that the HSP70 and PGF2S proteins are involved in parasite infectivity or infection patterns in the vertebrate host.

Prostaglandin synthesis has been reported to occur in metazoan and protozoan parasites in addition to mammals. However, the molecular mechanisms of prostaglandin production and their biological role in parasites have not been well elucidated [53]. High levels of prostaglandin F2-alpha (PGF_2α_) and PGF_2α_ synthase have been reported in *T*. *brucei* [57].

Interestingly, the overexpression of PGF_2α_ synthase increased the infectivity of the LbrC and LbrM isolates *in vitro* by improving parasite survival within host cells. Further investigation is needed to identify the mechanisms involved in the effect of PGF_2α_ synthase on parasite virulence.

It has been shown that PGF2S protein is present in the secretome of *L*. *(V*.*) braziliensis* [58] and the exosome of *L*. *(L*.*) donovani* [59]. According to the TDR Targets Database (tdrtargets.org), 13 putative antigenic epitopes in the PGF2S protein of *L*. *major* are responsible for 77.8% of its antigenicity, making it one of the most antigenic proteins produced by *L*. *major*. Using the same database, this protein was identified as having high potential as a drug target, with a 0.8 druggability index (range: 0.0 to 1.0). Therefore, *Lbr*PGF2S may represent a relevant target for studies of parasite-host interactions.

In conclusion, this study identified parasite-derived factors that contributed to the pathogenesis pattern of *L*. *braziliensis*. The genetic, proteomic and metabolomic results indicate that the inoculated population of parasites may contain a subpopulation of cells with a divergently expressed genome, leading to physiological differences that alter the modulatory response of the host.

## Supporting Information

S1 Fig Principal component analysis (PCA) plot of the metabolomic data revealing significant trends in the metabolic profiles of LbrM and LbrC. PCA models for the entire data set filtered according to their presence in at least 50% of the QCs and a coefficient of variance less than 30% in the QCs. A) HPLC-MS, 3 components (R^2^ = 0.383; Q^2^ = 0.040); B) CE-MS, 3 components (R^2^ = 0.389; Q^2^ = 0.006); and C) GC-MS, 4 components (R^2^ = 0.788, Q^2^ = 0.211). The LbrM and LbrC groups were obtained from seven replicates each. The QC group was obtained from four replicates.
(TIF)

S2 Fig Overexpression of LbrPGF2S in the LbrC and LbrM isolates. Western blotting analysis using an anti-*Lbr*PGF2S polyclonal antibody revealed the overexpression of *Lbr*PGF2S in the LbrC and LbrM transfectants. The anti-GAPDH antibody was used to monitor protein loading. The lower panel is a graphical representation of the densitometric analysis of the Western blot performed using Image J Software (National Institutes of Health, USA).
(TIFF)

S1 Table Read coverage for each chromosome of the LbrC^1^, LbrC^2^, LbrM^1^ and LbrM^2^ isolates by Illumina DNA sequencing. (DOCX)

S2 Table Number of mapped reads for each chromosome of the LbrC^1^, LbrC^2^, LbrM^1^ and LbrM^2^ isolates by Illumina DNA sequencing. (DOCX)

S3 Table Metabolites that were altered in the LbrC and LbrM isolates. (XLS)

S4 Table Proteins identified by MALDI-MS/MS that were differentially expressed in the LbrC and LbrM isolates. *The portion highlighted in grey represents proteins exhibiting differential expression patterns that were identical in the two patients.
(XLSX)
