# Supplementary figures and images for: Differential Gene Expression and Infection Profiles of Cutaneous and Mucosal Leishmania braziliensis Isolates from the Same Patient
Source: PLoS Negl Trop Dis. 2015 Sep 14;9(9):e0004018. doi: 10.1371/journal.pntd.0004018 (PMC4569073; doi:10.1371/journal.pntd.0004018)

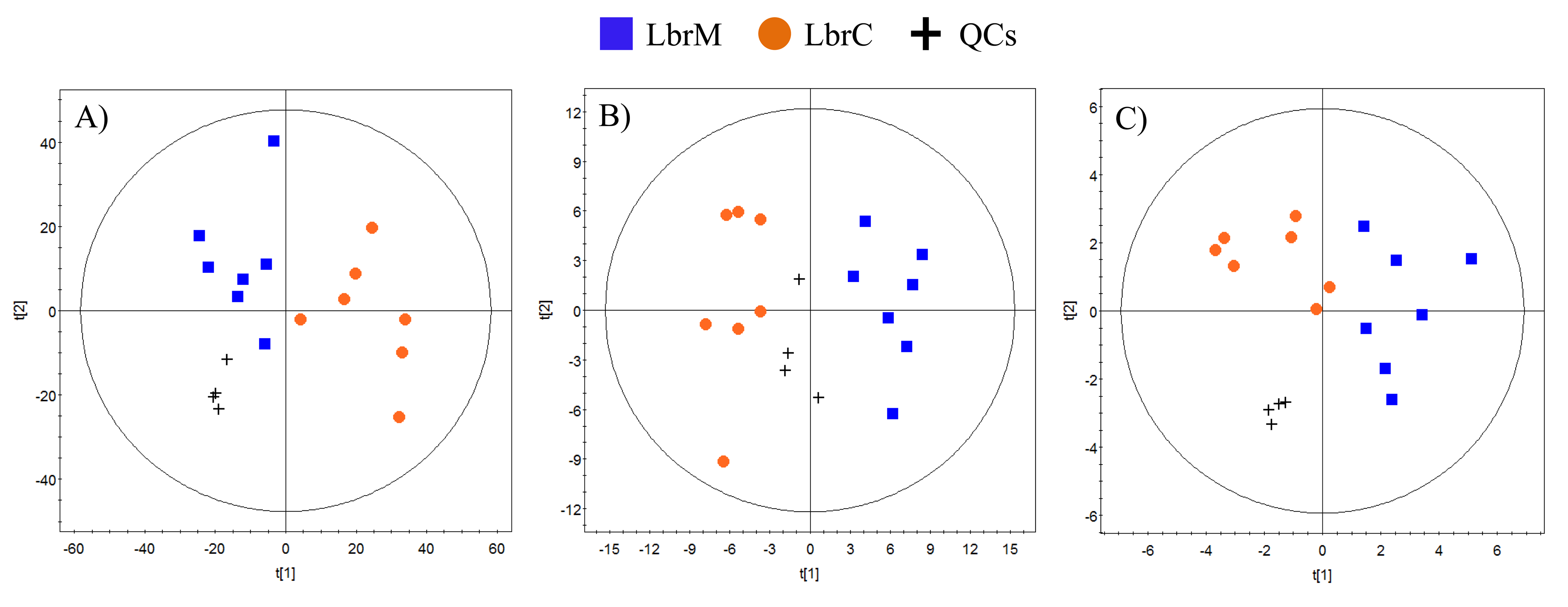

Supplement: S1 Fig — PCA models for the entire data set filtered according to their presence in at least 50% of the QCs and a coefficient of variance less than 30% in the QCs. A) HPLC-MS, 3 components (R2 = 0.383; Q2 = 0.040); B) CE-MS, 3 components (R2 = 0.389; Q2 = 0.006); and C) GC-MS, 4 components (R2 = 0.788, Q2 = 0.211). The LbrM and LbrC groups were obtained from seven replicates each. The QC group was obtained from four replicates. (TIF) [file pntd.0004018.s001.tif]

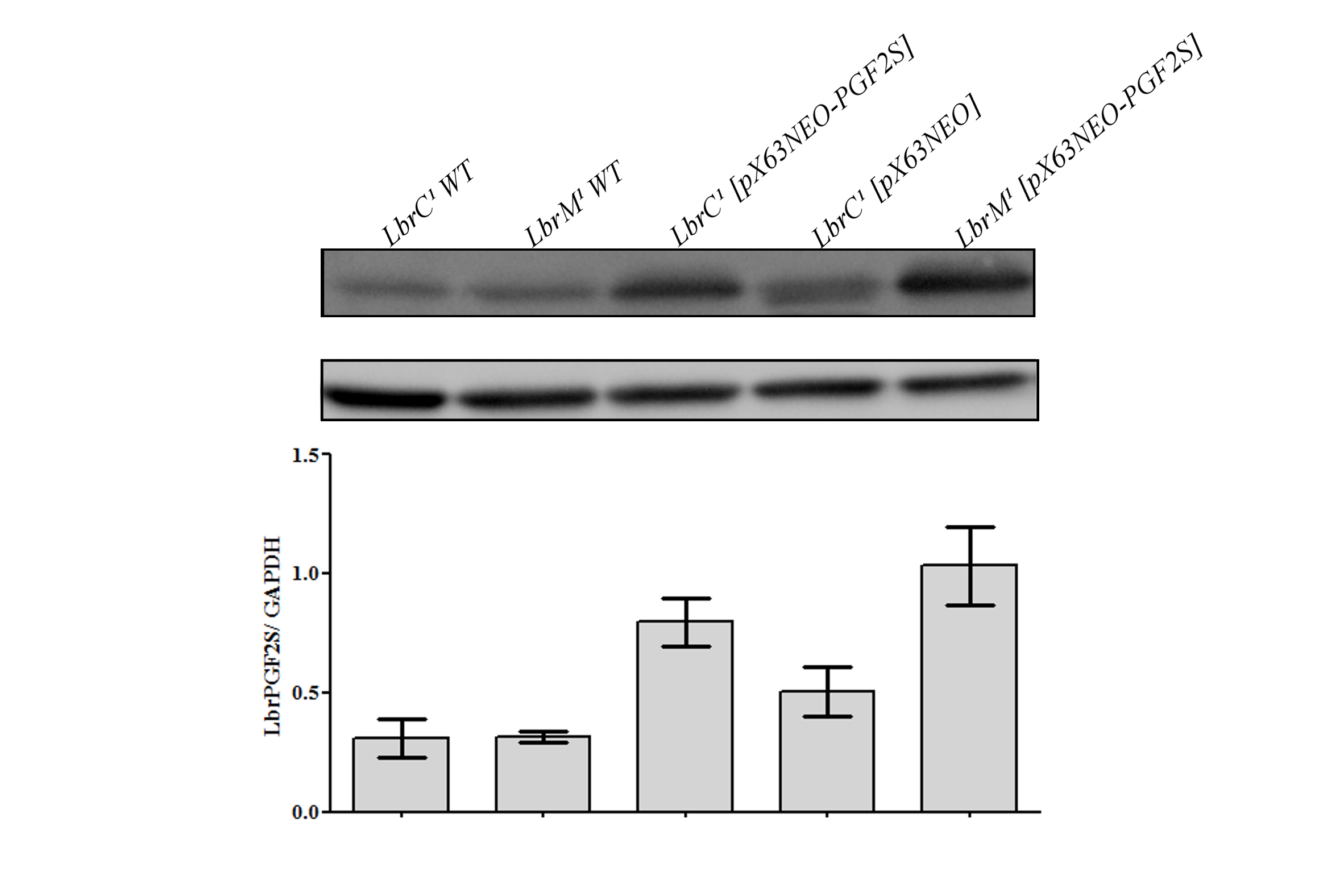

Supplement: S2 Fig — Western blotting analysis using an anti-LbrPGF2S polyclonal antibody revealed the overexpression of LbrPGF2S in the LbrC and LbrM transfectants. The anti-GAPDH antibody was used to monitor protein loading. The lower panel is a graphical representation of the densitometric analysis of the Western blot performed using Image J Software (National Institutes of Health, USA). (TIFF) [file pntd.0004018.s002.tiff]
