# Supplementary material for: Differential Gene Expression and Infection Profiles of Cutaneous and Mucosal Leishmania braziliensis Isolates from the Same Patient
Source: PLoS Negl Trop Dis. 2015 Sep 14;9(9):e0004018. doi: 10.1371/journal.pntd.0004018 (PMC4569073; doi:10.1371/journal.pntd.0004018)
Supplement: S1 Table — (DOCX) [file pntd.0004018.s003.docx]

| **Chromosome** | **LbrC^1^** | **LbrC^2^** | **LbrM^1^** | **LbrM^2^** |
| --- | --- | --- | --- | --- |
| 1 | 4.36 | 6.19 | 3.01 | 3.57 |
| 2 | 4.74 | 6.70 | 3.36 | 3.93 |
| 3 | 5.33 | 7.41 | 3.63 | 4.36 |
| 4 | 5.31 | 9.10 | 3.66 | 4.42 |
| 5 | 5.24 | 7.43 | 3.70 | 4.37 |
| 6 | 6.86 | 9.55 | 4.65 | 5.37 |
| 7 | 5.90 | 8.10 | 3.96 | 4.95 |
| 8 | 5.96 | 8.61 | 4.36 | 5.13 |
| 9 | 5.54 | 7.72 | 3.79 | 4.54 |
| 10 | 5.45 | 7.43 | 3.63 | 4.46 |
| 11 | 6.45 | 8.57 | 4.03 | 5.36 |
| 12 | 8.80 | 11.75 | 5.67 | 7.52 |
| 13 | 6.24 | 8.63 | 4.09 | 5.27 |
| 14 | 6.34 | 8.47 | 4.12 | 5.40 |
| 15 | 6.01 | 8.29 | 4.05 | 5.09 |
| 16 | 6.30 | 8.80 | 4.42 | 5.26 |
| 17 | 6.33 | 8.56 | 4.26 | 5.23 |
| 18 | 6.70 | 9.11 | 4.36 | 5.83 |
| 19 | 6.08 | 8.30 | 3.91 | 5.26 |
| 20 | 7.13 | 9.45 | 4.58 | 6.13 |
| 21 | 5.97 | 8.26 | 4.02 | 5.02 |
| 22 | 6.37 | 8.48 | 4.14 | 5.46 |
| 23 | 6.96 | 9.31 | 4.49 | 5.87 |
| 24 | 5.87 | 8.13 | 4.05 | 4.93 |
| 25 | 6.18 | 8.61 | 4.09 | 5.39 |
| 26 | 6.36 | 8.57 | 4.14 | 5.35 |
| 27 | 6.72 | 8.94 | 4.32 | 5.72 |
| 28 | 6.86 | 9.31 | 4.43 | 5.87 |
| 29 | 6.79 | 9.21 | 4.42 | 5.79 |
| 30 | 6.82 | 9.16 | 4.33 | 5.84 |
| 31 | 11.62 | 15.52 | 7.48 | 10.05 |
| 32 | 7.26 | 9.63 | 4.63 | 6.24 |
| 33 | 7.10 | 9.49 | 4.50 | 6.08 |
| 34 | 7.50 | 9.96 | 4.70 | 6.53 |
| 35 | 6.81 | 9.18 | 4.43 | 5.99 |
