# Supplementary material for: Differential Gene Expression and Infection Profiles of Cutaneous and Mucosal Leishmania braziliensis Isolates from the Same Patient
Source: PLoS Negl Trop Dis. 2015 Sep 14;9(9):e0004018. doi: 10.1371/journal.pntd.0004018 (PMC4569073; doi:10.1371/journal.pntd.0004018)
Supplement: S2 Table — (DOCX) [file pntd.0004018.s004.docx]

| **Chromosome** | **LbrC^1^** | **LbrC^2^** | **LbrM^1^** | **LbrM^2^** |
| --- | --- | --- | --- | --- |
| 1 | 8,332 | 11,829 | 5,752 | 6,831 |
| 2 | 11,142 | 15,760 | 7,906 | 9,241 |
| 3 | 14,524 | 20,184 | 9,883 | 11,887 |
| 4 | 17,797 | 30,516 | 12,262 | 14,835 |
| 5 | 17,634 | 25,035 | 12,458 | 14,713 |
| 6 | 26,992 | 37,580 | 18,294 | 21,144 |
| 7 | 25,147 | 34,545 | 16,880 | 21,085 |
| 8 | 21,357 | 30,871 | 15,610 | 18,366 |
| 9 | 22,924 | 31,971 | 15,692 | 18,810 |
| 10 | 25,239 | 34,426 | 16,815 | 20,641 |
| 11 | 29,420 | 39,097 | 18,375 | 24,454 |
| 12 | 23,950 | 31,991 | 15,419 | 20,468 |
| 13 | 27,391 | 37,867 | 17,950 | 23,114 |
| 14 | 27,512 | 36,743 | 17,884 | 23,407 |
| 15 | 26,257 | 36,208 | 17,684 | 22,223 |
| 16 | 32,408 | 45,259 | 22,729 | 27,061 |
| 17 | 31,255 | 42,238 | 21,039 | 25,816 |
| 18 | 36,767 | 49,984 | 23,933 | 31,995 |
| 19 | 29,590 | 40,425 | 19,028 | 25,619 |
| 20 | 135,998 | 180,344 | 87,467 | 116,960 |
| 21 | 31,149 | 43,145 | 20,986 | 26,202 |
| 22 | 30,876 | 41,109 | 20,096 | 26,469 |
| 23 | 42,412 | 56,733 | 27,339 | 35,751 |
| 24 | 36,505 | 50,568 | 25,179 | 30,654 |
| 25 | 39,909 | 55,582 | 26,391 | 34,792 |
| 26 | 43,664 | 58,903 | 28,476 | 36,736 |
| 27 | 57,396 | 76,428 | 36,903 | 48,915 |
| 28 | 56,717 | 76,942 | 36,573 | 48,550 |
| 29 | 56,876 | 77,094 | 36,972 | 48,505 |
| 30 | 66,407 | 89,139 | 42,115 | 56,814 |
| 31 | 134,721 | 179,929 | 86,729 | 116,505 |
| 32 | 80,701 | 107,000 | 51,470 | 69,347 |
| 33 | 74,860 | 100,067 | 47,434 | 64,129 |
| 34 | 109,590 | 145,626 | 68,655 | 95,495 |
| 35 | 131,152 | 176,966 | 85,301 | 115,362 |
